# Benign Osteoblastoma Involving Maxilla: A Case Report and Review of the Literature

**DOI:** 10.1155/2012/351241

**Published:** 2012-04-23

**Authors:** K. Bokhari, M. S. Hameed, M. Ajmal, Rafi A. Togoo

**Affiliations:** College of Dentistry, King Khalid University, Abha 3263, Saudi Arabia

## Abstract

*Background*. Osteoblastoma is a rare benign tumor. This tumor is characterized by osteoid and bone formation with the presence of numerous osteoblasts. The lesion is more frequently seen in long bones and rarely involves maxilla and mandible. Due to its clinical and histological similarity with other bone tumors such as osteoid osteoma and fibro-osseous lesions, osteoblastoma presents a diagnostic dilemma. *Case Report*. Very few cases of osteoblastomas involving maxillofacial region have been reported in the literature. This case report involves osteoblastoma involving right maxilla in an 18-year-old male patient. Following detailed clinical examination, radiological interpretation, and histopathological diagnosis, surgical excision was performed. The patient was followed up for a period of 3 years and was disease free. *Summary and Conclusion*. Benign osteoblastoma involving jaw bones is a rare tumor. There is a close resemblance of this tumor with other lesions such as fibro-osseous lesions and odontogenic tumors and thus faces a diagnostic challenge. Surgical excision with a long-term follow-up gives good prognosis to this lesion—Benign Osteoblastoma.

## 1. Introduction

Benign osteoblastoma is a rare tumor of bone representing less than 1% of all tumors in the maxillofacial region [1]. It usually occurs in young adults, with a mean age of 20 years, and primarily involves the vertebral column, long bones, small bones of hands and feet (metacarpal and metatarsal), and facial bones including the jaw [2]. Histologically, this tumor is characterized by osteoid and woven bone deposition and abundant osteoblasts that are frequently in close association with newly formed bone [3]. There are two main clinicopathological entities of osteoblastoma: the benign form, which grows slowly over many years and has a well-defined sclerotic margin, is fairly well vascularized with a mild inflammatory response and an aggressive form [4]. The aggressive form of osteoblastomas exhibits locally aggressive behavior with a propensity to recur and has atypical histopathological features, often making differentiation from low-grade osteosarcoma difficult [5]. Osteoblastomas can also be classified as cortical, medullary, and periosteal types depending on which component of the bone is involved [6]. Those involving the jaws are either medullary or periosteal with the cortical variant commonly seen in the extragnathic sites [7].

Due to a wide spectrum of bone lesions reported in the literature with very close clinical, radiological, and histological interrelations, these tumors pose a diagnostic challenge. This may cause relevant problems with the differential diagnosis in view of the tumor's rarity, ambiguous clinicoradiologic presentation, and histologic features, which sometimes resemble osteosarcoma [3]. A definitive diagnosis and clear treatment plan can only be formulated after correlating all the three components (i.e., clinical, histological, and radiological features). We report an interesting and rare case of benign osteoblastoma involving left maxilla treated by surgical excision in a 18-year-old male patient. 

## 2. Case Report

**Figure 1:**
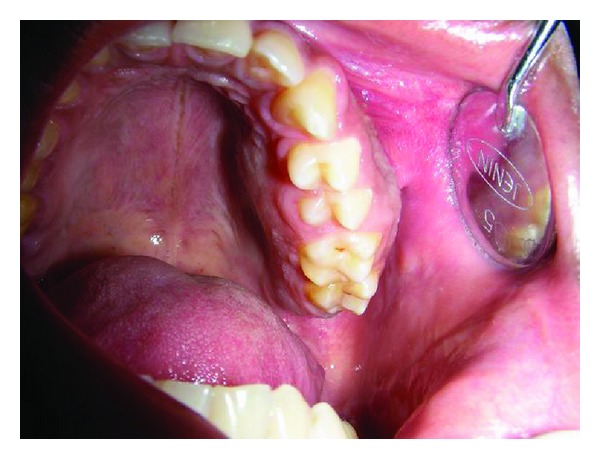
Lesion extension intraorally.

 A 18-year-old male patient reported to our center with a chief complaint of pain and swelling of the left maxilla since 6 months. On extraoral examination, there was a diffuse bony swelling of the left maxilla which was slightly tender on palpation. Intraoral examination revealed bicortical expansion of the left maxillary alveolar ridge with the swelling extending from left first premolar to the first molar which was roughly elliptical in outline and nontender (Figure 1). The swelling was hard in consistency and measured approximately 3∗2 centimeters in greatest dimension. No mobility of the teeth in the region was elicited and there was obliteration of the buccal vestibule. Mucosa over the swelling was normal with absence of any ulcerations or sinus discharge. There were no other secondary changes involved like paresthesia or cervical lymphadenopathy.

**Figure 2:**
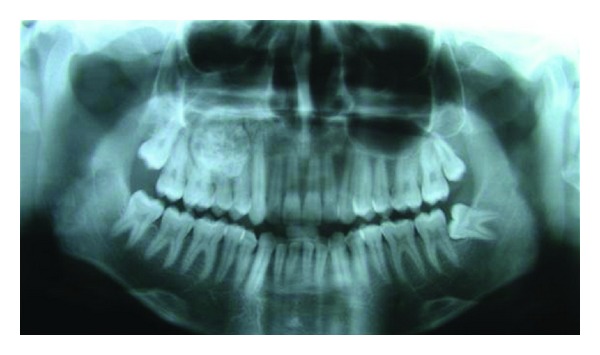
Orthopantomogram showing the lesion.

**Figure 3:**
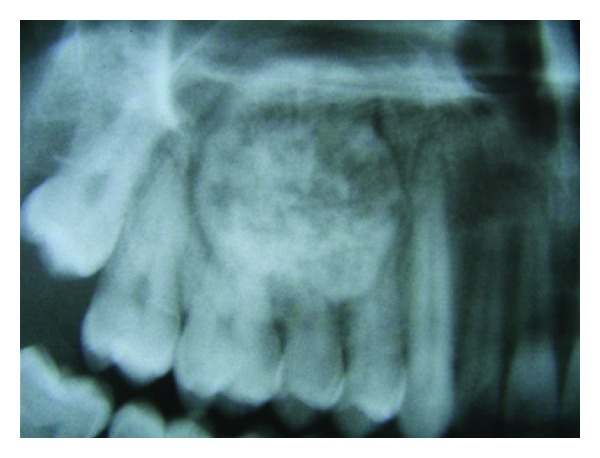
Radiograph showing the extent of lesion.

 Radiographic examination revealed a well-circumscribed, radio-opaque lesion mixed with areas of radiolucency. The lesion was roughly round to oval in outline and measured around 3∗3.5 cm in diameter. The periphery of the lesion was surrounded by a well-defined radiolucent rim (Figure 2). There was no reactive bone forming rim at the periphery. Radiographically, the lesion was extending from distal surface of canine root anteriorly to the mesial surface of second molar posteriorly. Superiorly, the lesion was invading into the maxillary sinus and the inferior extent was almost till the alveolar ridge (Figure 3).

**Figure 4:**
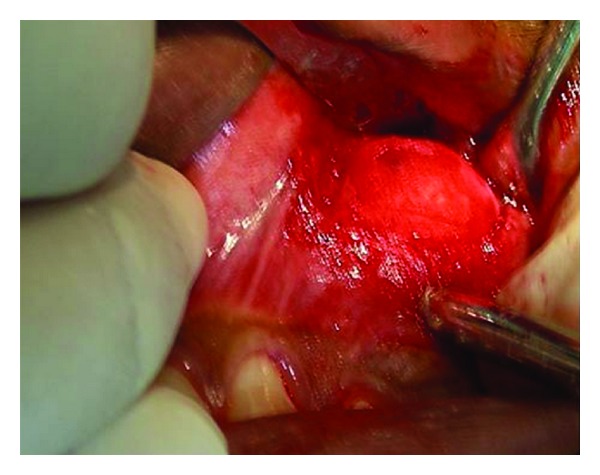
Intra-operative view of the tumor.

**Figure 5:**
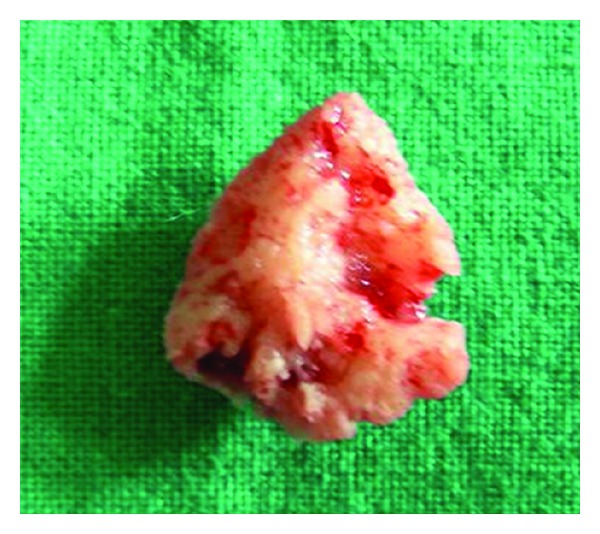
Excised specimen.

**Figure 6:**
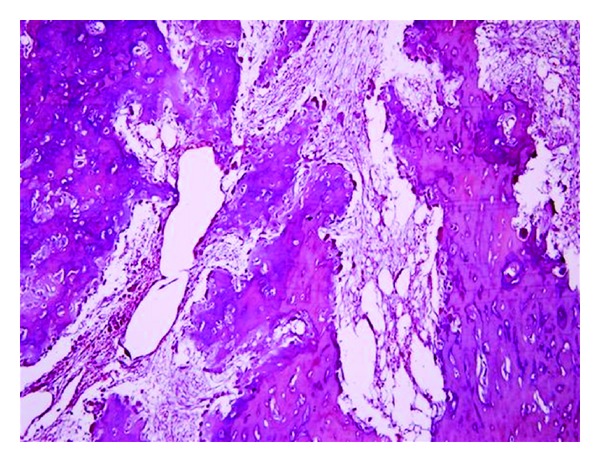
Histopathological picture of the lesion.

 Incisional biopsy was performed under local anesthesia. Access to the lesion was gained through a buccal crevicular incision. A diagnosis of benign bone tumor was then made based on the histological, radiological, and clinical findings. Complete excision of the tumor mass was planned under general anesthesia. Under strict aseptic precautions, complete excision of the tumor was performed through a buccal vestibular incision extending from canine to the second molar (Figure 4). Intraoperatively, the tumor was not very vascular and as it was well circumscribed which facilitated in toto excision (Figure 5). Complete hemostasis was achieved and primary closure was performed. The specimen was sent for histopathological examination which confirmed the diagnosis of benign osteoblastoma.  Figure 6 shows vascularized fibrous connective tissue with bony trabeculae lined by numerous osteoblasts and scattered osteoclasts. Epitheloid like osteoblasts are present in some areas. Resorption of the trabeculae by multinucleated osteoclasts and prominent reversal lines are observed.

 There were no specific complaints postoperatively, and the patient was discharged 5 days postoperatively. The patient was disease free without any recurrence during the follow-up period.

## 3. Discussion

Benign osteoblastoma is a rare osteoblastic tumor of bone [8]. This benign neoplasm of bone is characterized by proliferation of osteoblasts forming trabeculae set in a vascularized fibrous connective tissue stroma [9]. The etiopathogenesis of this lesion is varied and widely debated in the literature with no clear consensus on its origin. Jaffe and Lichtenstein stated this lesion to be a true neoplasm of osteoblastic derivation [10, 11]. Trauma, inflammation, abnormal local response of the tissues to injury, and local alteration in bone physiology are few of the other reasons cited in the literature pertaining to the etiology of this tumor.

Due to its rarity and close resemblance with other bony tumors of the jaws, osteoblastoma presents a diagnostic challenge at the first clinical presentation. Differential diagnosis may include fibro-osseous lesions, bone tumors, and odontogenic tumors. Osteoblastoma itself has been subclassified into different variants based on their aggressive nature and histopathological findings. Capodiferro et al., in their clinicopathologic study of four cases involving osteoblastoma of the mandible, elaborated on two variants, namely, the so-called toxic osteoblastoma and the aggressive osteoblastoma with each one having its specific characteristics [3]. Osteosarcoma is another entity which needs to be considered while dealing with osteoblastoma. Distinction of osteoblastoma from osteosarcoma is based on the absence of atypical mitotic figures, cellular pleomorphism, neoplastic cartilage, and permeative growth into adjacent bone tissue [12].

Benign osteoblastoma clinically presents mainly with slight pain, swelling, and expansion of bone cortex. They have a limited growth potential and typically do not exceed 4 cm in diameter [2]. Radiographic picture of osteoblastoma is not very consistent and varies from case to case depending on the duration [12]. A combination of radiopaque and radiolucent patterns, depending on the degree of calcification and absence of perilesional sclerotic border, is a general radiographic finding for benign osteoblastomas. Histologically, osteoblastoma is a bone-forming tumor characterized by osteoid and woven bone deposition and abundant osteoblasts that are frequently in close association with newly formed bone [3]. The widely accepted treatment for benign osteoblastomas is surgical excision. A more conservative approach of surgical curettage has been suggested in the literature [6]. Nowparast et al. stated that benign osteoblastoma has a good prognosis and is best treated by curettage or conservative surgical excision [13]. Woźniak et al., in their case report involving malignant transformation of an osteoblastoma of the mandible, suggested complete resection with the margins located in the normal tissues as the treatment of choice and recommend additional radiotherapy and/or chemotherapy in more aggressive cases [4]. Considering the recurrence rate of 13.6% for benign osteoblastomas [2, 3], surgical excision of the entire tumor would be the preferred treatment as curettage would lead to recurrence. Radiotherapy and more aggressive resection should be restricted to the aggressive variants of osteoblastoma. In this case report, the patient was successfully treated by surgical excision and had no incidence of recurrence. 

## 4. Conclusion

1. Benign osteoblastoma involving jaw bones is a rare tumor.
2. There is a close resemblance of this tumor with other lesions such as fibro-osseous lesions and odontogenic tumors and thus faces a diagnostic challenge.
3. Surgical excision with a long-term follow-up gives good prognosis to this lesion—Benign Osteoblastoma.
